# Sex Differences in Psychotropic Drug Exposure and Safety: A Systematic Review Toward Personalized Dosing Strategies

**DOI:** 10.3390/jpm16040189

**Published:** 2026-03-31

**Authors:** Maria Puntarello, Giuseppe Davide Albano, Stefania Zerbo, Ginevra Malta, Antonina Argo

**Affiliations:** Department of Health Promotion, Mother and Childcare, Internal Medicine and Medical Specialties (PROMISE), University of Palermo, Piazza delle Cliniche 2, 90127 Palermo, Italy; maria.puntarello@community.unipa.it (M.P.); giuseppedavide.albano@unipa.it (G.D.A.); stefania.zerbo@unipa.it (S.Z.); ginevra.malta@unipa.it (G.M.)

**Keywords:** sex differences, pharmacokinetics, personalized medicine, ADR

## Abstract

**Background**: Biological sex contributes to variability in drug metabolism, receptor sensitivity, and susceptibility to adverse drug reactions (ADRs). Despite this, dosing recommendations for selective serotonin reuptake inhibitors (SSRIs) and second-generation antipsychotics (SGAs) are still largely sex-neutral. This systematic review examines sex-related differences in pharmacokinetics (PK), pharmacodynamics (PD), and safety outcomes, with the aim of clarifying their potential implications for personalized psychopharmacology. **Methods**: A systematic search of PubMed was conducted for studies published between January 2010 and March 2026. The strategy combined MeSH terms and free-text keywords related to SSRIs, SGAs, sex differences, pharmacokinetics, pharmacodynamics, and ADRs. Two independent reviewers performed study selection and data extraction. Studies reporting sex-stratified PK, PD, or safety outcomes in humans were included. Owing to methodological heterogeneity, results were synthesized narratively. **Results**: Twenty-seven studies met the inclusion criteria. Overall, the evidence indicates clinically meaningful sex-related differences in psychotropic drug exposure and response. Women more frequently exhibited higher dose-adjusted serum concentrations, particularly for risperidone and some SSRIs, with age-related increases more evident in females. Pharmacodynamic findings suggest that women may reach comparable dopamine D2 receptor occupancy at lower olanzapine doses. Pharmacovigilance analyses revealed sex-specific adverse event patterns, including greater reporting of endocrine-related effects and QT prolongation in women. **Conclusions**: Sex influences psychotropic drug exposure, pharmacodynamic sensitivity, and safety profiles in ways that may be clinically relevant. Integrating sex-aware considerations into dosing strategies could improve therapeutic precision and reduce adverse outcomes, reinforcing the importance of sex as a key variable in personalized psychiatric care.

## 1. Introduction

Pharmacological research has historically been shaped by an androcentric paradigm, in which male subjects have been disproportionately represented in both preclinical and clinical studies [1]. This imbalance has been driven by concerns related to hormonal variability and reproductive risk in women, as well as by the assumption that findings derived from male populations could be generalized across sexes. As a consequence, sex-specific biological variability has often been overlooked in drug development, regulatory processes, and clinical prescribing practices [2].

Although regulatory initiatives—such as the inclusion mandates introduced in the 1990s—have improved female participation in clinical trials, the consideration of sex as a biological variable remains inconsistent [3]. In the United States, regulatory attention to sex inclusion began with the NIH Revitalization Act of 1993, which mandated the inclusion of women in federally funded clinical research, followed by FDA guidelines encouraging the evaluation of sex differences in drug development [4]. More recently, the NIH policy on sex as a biological variable (SABV), introduced in 2016, has further emphasized the need to integrate sex-based analyses across all stages of biomedical research. Meanwhile, in Europe, regulatory frameworks—primarily shaped by the European Medicines Agency—have progressively acknowledged the importance of subgroup analyses, including sex differences [5]. However, unlike the United States, no uniformly binding requirement exists for the systematic integration of sex as a biological variable across all clinical studies, resulting in heterogeneous implementation [6]. A substantial proportion of studies still fail to perform sex-stratified analyses or treat sex merely as a covariate, rather than as a determinant of pharmacological response [7]. Even in fields where sex differences are known to be relevant, such as neuropsychopharmacology, relatively few studies are specifically designed to evaluate differential drug effects between males and females, and many lack sufficient statistical power to detect such differences [8].

The clinical implications of this gap are increasingly evident. Women experience adverse drug reactions nearly twice as often as men, a disparity that has been consistently associated with sex-related differences in pharmacokinetics, including higher systemic drug exposure, reduced clearance, and prolonged elimination times [9]. Importantly, these differences cannot be fully explained by body weight alone, but instead reflect a complex interplay of biological factors, including differences in body composition, plasma protein binding, enzymatic activity, renal function, and hormonal regulation [10].

In addition to pharmacokinetic variability, sex differences also extend to pharmacodynamic processes, particularly within the central nervous system. Experimental and neuroimaging studies suggest that males and females may exhibit distinct neural responses to pharmacological agents, as well as differences in treatment efficacy and side-effect profiles [11]. Despite this growing body of evidence, most medications—including psychotropic drugs—continue to be prescribed according to sex-neutral dosing strategies, with only limited consideration of potential sex-specific optimization [12].

Collectively, these observations highlight a persistent disconnect between emerging scientific evidence and current clinical practice. Addressing sex-related variability in drug response is therefore essential for improving therapeutic precision [13]. Incorporating sex as a key biological variable represents a fundamental step toward the development of personalized medicine, with the potential to enhance efficacy, reduce adverse outcomes, and optimize pharmacological treatment strategies [14].

Psychiatric disorders remain among the leading causes of disability worldwide. SSRIs and second-generation antipsychotics are widely prescribed across age groups and diagnostic categories. Although their efficacy is well established, treatment response is often variable, and adverse drug reactions frequently compromise tolerability and long-term adherence [15]. Growing evidence suggests that biological sex represents an important source of interindividual variability in psychotropic drug exposure, therapeutic response, and safety. Sex-related differences in pharmacokinetics (PK) stem from variations in body composition, plasma protein binding, cytochrome P450 activity, renal elimination, and hormonal regulation [16]. Large therapeutic drug monitoring datasets indicate that dose-adjusted serum concentrations of several antipsychotics increase with age in both sexes, but this rise appears more pronounced in women, particularly for risperidone [17]. Population pharmacokinetic modeling studies further suggest that men may exhibit higher apparent clearance of olanzapine compared with women. Although CYP2D6 polymorphisms account for substantial variability in risperidone exposure, sex remains an independent contributor [18]. Beyond systemic exposure, pharmacodynamic (PD) mechanisms may play a decisive role. Modeling studies indicate that women may achieve comparable dopamine D2 receptor occupancy at lower antipsychotic doses, suggesting greater central dopaminergic sensitivity [19].

Experimental work has also demonstrated sex-specific neurophysiological responses following olanzapine administration, including differences in sleep architecture despite similar plasma levels [20]. These observations support the idea that receptor-level dynamics and neuroendocrine modulation may differ between sexes. Safety data further reinforce this pattern. Pharmacovigilance analyses report a higher proportion of ADRs among women treated with SSRIs, especially dose-related and commonly occurring reactions [21].

Large-scale database analyses of antipsychotic-associated adverse events also reveal sex-dependent reporting patterns [22]. In addition, women are known to have a higher susceptibility to drug-induced long QT syndrome, likely reflecting sex-specific differences in cardiac repolarization reserve influenced by estrogen [23]. Endocrine adverse effects, including hyperprolactinemia, are also more frequently reported in female patients. Age appears to amplify these differences. Older women, in particular, show higher concentration-to-dose ratios for several antipsychotics, highlighting the clinical importance of age–sex interactions [24]. Nevertheless, prescribing practices remain largely sex-neutral, even in vulnerable populations such as nursing home residents with dementia, where sex-related differences in exposure have been documented [25].

Although prior reviews have addressed sex differences in psychopharmacology, evidence remains fragmented across PK, PD, and safety domains. Moreover, many clinical trials are not sufficiently powered for robust sex-stratified pharmacometric analyses [26].

In a field characterized by narrow therapeutic windows and potentially serious adverse effects, understanding whether sex-related variability justifies tailored dosing strategies is of clear clinical importance. This systematic review, therefore, aims to comprehensively evaluate sex-specific differences in pharmacokinetics, pharmacodynamics, and adverse drug reactions associated with SSRIs and SGAs, and to explore their implications for sex-informed and personalized dosing approaches.

## 2. Materials and Methods

### 2.1. Study Design

This systematic review was conducted in accordance with PRISMA guidelines. The PRISMA 2020 checklist is provided in the Supplementary Materials. In a preliminary phase, the research question was developed based on the current scientific context regarding sex-related differences in pharmacokinetics, pharmacodynamics, and drug safety. This conceptual framework was informed by an initial exploratory analysis of key literature addressing sex differences in clinical pharmacology and neuropsychopharmacology. These articles were used to identify knowledge gaps and refine the study objective but were not included in the systematic review synthesis. The review protocol was prospectively registered on the Open Science Framework (OSF) and is currently available in view-only mode due to embargo restrictions (https://osf.io/qhgu8/overview?view_only=fd6a37c647ac4a0dbdcdd77175591ca8) (accessed on 1 March 2026). The objective was to synthesize available evidence on sex-related differences in PK, PD, and ADRs associated with SSRIs and SGAs, focusing on their potential relevance for personalized psychopharmacology.

### 2.2. Data Sources and Search Strategy

A systematic search of PubMed was performed in February 2026, covering studies published from 1 January 2010 to the search date, to observe the most recent articles, without language restrictions. The strategy combined MeSH terms and free-text keywords relating to drug classes (SSRIs and SGAs), sex-related variables, and pharmacological or safety outcomes. Boolean operators were used to ensure both sensitivity and specificity. The literature search was conducted in the PubMed database using a structured strategy based on MeSH terms and relevant keywords. No language restrictions were applied during the search process. All studies ultimately included in the qualitative synthesis were published in English.

The three conceptual domains—drug class, sex variables, and outcomes—were combined using “AND” to restrict retrieval to studies addressing all relevant components simultaneously.

The final search string was:

(“Selective Serotonin Reuptake Inhibitors”[Mesh] OR “SSRI”[Title/Abstract] OR “selective serotonin reuptake inhibitor”[Title/Abstract] OR “Antipsychotic Agents”[Mesh] OR “atypical antipsychotic”[Title/Abstract] OR “second-generation antipsychotic”[Title/Abstract]) AND (“Sex Factors”[Mesh] OR “sex differences”[Title/Abstract] OR “sex-specific”[Title/Abstract] OR “gender differences”[Title/Abstract]) AND (“Drug-Related Side Effects and Adverse Reactions”[Mesh] OR “adverse event” [Title/Abstract] OR “adverse drug reaction” [Title/Abstract] OR “safety” [Title/Abstract] OR “toxicity” [Title/Abstract] OR “pharmacokinetic” [Title/Abstract] OR “pharmacodynamic”[Title/Abstract]) AND “Humans”[Mesh] AND (“2010/01/01”[Date-Publication]: “3000”[Date-Publication]).

Reference lists of relevant articles were also manually screened to identify additional eligible studies. The complete list of the 120 records identified through the PubMed search is provided in the Supplementary Materials.

### 2.3. Study Selection and Eligibility Criteria

Two reviewers independently screened titles and abstracts. Potentially relevant articles were assessed in full text. Disagreements were resolved through discussion and consensus. Studies were eligible if they investigated SSRIs or SGAs in human populations and reported sex-stratified outcomes or explicitly analyzed sex as a biological variable. Eligible outcomes included pharmacokinetic parameters, pharmacodynamic measures, and adverse drug reactions or safety endpoints. Animal studies, case reports, editorials, and narrative reviews were excluded from the primary synthesis, although reviews and meta-analyses were used for contextual interpretation.

### 2.4. Data Extraction and Synthesis

Data extraction was conducted independently by two reviewers using a standardized form. Extracted information included study design, population characteristics, drug exposure, PK and PD parameters, safety outcomes, and reported sex-related differences.

Given the heterogeneity in study designs and outcome reporting, quantitative meta-analysis was not feasible. Findings were therefore synthesized narratively and organized by thematic domains: pharmacokinetics, pharmacodynamics, adverse drug reactions, and age–sex interactions, with attention to their implications for personalized dosing.

## 3. Results

**Figure 1:**
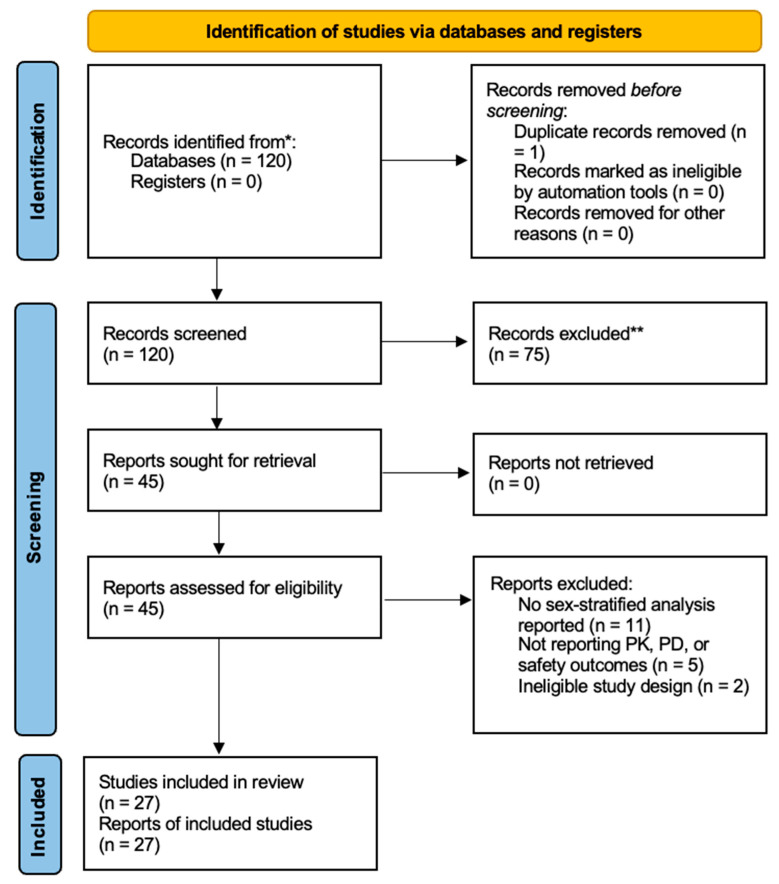
PRISMA flow diagram. * Records identified from PubMed database. ** Records excluded after title and abstract screening due to lack of sex-stratified analysis, absence of relevant pharmacokinetic, pharmacodynamic or safety outcomes, or not meeting the inclusion criteria. Duplicate records were removed prior to screening; therefore, the 120 records represent the unique records screened. Duplicate records were removed prior to screening; therefore, the 120 records represent the unique records screened.

The search yielded 120 records. After screening, 75 were excluded at the title and abstract level. Forty-eight full-text articles were assessed; 20 were excluded for lack of sex-specific analyses or relevant outcomes. Twenty-seven studies were ultimately included in the qualitative synthesis (Figure 1).

The literature search identified 120 records, of which 27 studies met the inclusion criteria following title, abstract, and full-text screening. The search strategy did not include language restrictions; however, all studies that met the inclusion criteria and were ultimately analyzed were published in English.

The included studies comprised therapeutic drug monitoring datasets, population pharmacokinetic and pharmacodynamic modeling investigations, pharmacovigilance database analyses, and observational cohort studies conducted in both general and elderly populations. Together, these investigations provide converging evidence across exposure, receptor-level effects, and safety outcomes. Across pharmacokinetic studies, sex-dependent differences in systemic drug exposure were consistently observed, although their magnitude varied by compound. Large-scale therapeutic drug monitoring analyses demonstrated that dose-adjusted serum concentrations of several antipsychotics increased with age in both sexes but were generally higher in females, particularly for risperidone, where concentration-to-dose ratios rose markedly in older women [27]. Additional serum-level investigations confirmed that age and sex jointly influence exposure variability across multiple agents. Population pharmacokinetic modeling of olanzapine suggested higher apparent clearance in males compared with females, supporting sex-related differences in drug disposition [28]. Although CYP2D6 polymorphisms accounted for substantial variability in risperidone exposure, sex remained an independent contributor to concentration differences, indicating that pharmacogenetic and biological sex factors act in parallel rather than in isolation [29]. For SSRIs, pharmacovigilance and clinical data suggested that women more frequently experienced dose-related adverse reactions, indirectly supporting exposure-related variability [30]. Pharmacodynamic evidence indicated that sex-related variability extends beyond systemic exposure. Modeling studies of dopamine D2 receptor occupancy demonstrated that females may achieve comparable receptor blockade at lower doses of olanzapine compared with males, suggesting increased central sensitivity [30]. Experimental investigations in healthy volunteers revealed sex-specific neurophysiological responses to olanzapine, including differential modulation of slow-wave sleep despite similar plasma concentrations [30,31]. These findings support the hypothesis that sex-dependent receptor dynamics and neuroendocrine modulation contribute to divergent pharmacodynamic responses independent of plasma exposure alone. Sex-specific patterns were also evident in safety outcomes. Analyses of the Duch national pharmacovigilance systems reported higher proportions of dose-related and commonly occurring adverse drug reactions in women treated with SSRIs, while large-scale text-mining and spontaneous reporting database studies identified sex-dependent reporting signals across multiple adverse event categories for antipsychotics [32,33,34,35,36,37]. Women were consistently recognized as having increased susceptibility to drug-induced long QT syndrome, a vulnerability attributed to sex-specific differences in cardiac repolarization reserve and hormonal modulation [33]. Endocrine-related adverse effects, including hyperprolactinemia, were more frequently reported among female antipsychotic users [32]. Observational cohort studies conducted in older and institutionalized populations further demonstrated sex-related differences in antipsychotic exposure and continuation patterns, indicating that biological vulnerability and prescribing practices may interact to shape safety outcomes [34]. However, not all outcomes demonstrated uniform sex effects. For example, analyses based on the JADER pharmacovigilance database (Ebina et al.) identified sex-dependent signals for specific adverse events such as dystonia, but these were not consistent across different drugs or outcome categories, supporting drug- and event-specific variability rather than universal sex-dependent patterns [31]. Age emerged as a critical modifier of sex-related differences. Older adults, particularly women, exhibited disproportionately elevated concentration-to-dose ratios across multiple antipsychotics [27], reinforcing the clinical relevance of combined age–sex interactions in concentration-dependent toxicity. Overall, the integrated analysis of pharmacokinetic, pharmacodynamic, and safety data indicates that sex influences systemic exposure, receptor-level drug sensitivity, and adverse reaction susceptibility in compound-specific ways. Overall, the 27 included studies comprised 18 primary investigations and 9 secondary sources of evidence. Primary studies provided original data on pharmacokinetics, pharmacodynamics, therapeutic drug monitoring, pharmacovigilance signals, genetic associations, and prescribing patterns, whereas secondary studies offered integrative and mechanistic interpretations of sex-related variability in psychotropic drug response and adverse reactions. The characteristics and key findings of primary investigations are summarized in Table 1, while secondary evidence contextualizing these findings is presented in Table 2. Together, these data demonstrate that sex influences psychotropic drug exposure, receptor-level sensitivity, and safety profiles in compound-specific and age-dependent ways (Table 1 and Table 2).

## 4. Discussion

This systematic review integrates pharmacokinetic, pharmacodynamic, and pharmacovigilance evidence to evaluate whether biological sex meaningfully influences psychotropic drug exposure and safety. The findings suggest that sex is not merely a demographic descriptor but a biologically relevant determinant of variability in systemic drug concentrations, receptor-level effects, and adverse reaction susceptibility. However, the magnitude and clinical implications of these differences vary across compounds and contexts. From a pharmacokinetic perspective, the most consistent signal emerging from therapeutic drug monitoring studies is the tendency toward higher dose-adjusted serum concentrations in females, particularly with advancing age [27]. These differences likely reflect multifactorial mechanisms, including lower average body weight, higher body fat composition, sex-dependent hepatic enzyme activity, and age-related changes in metabolic clearance. Nevertheless, genetic polymorphisms—such as CYP2D6 metabolizer status—often exert a stronger influence on exposure variability than sex alone [29]. This suggests that sex and pharmacogenetics should not be considered competing variables but rather interacting components within a broader precision-medicine framework. Importantly, pharmacodynamic findings indicate that exposure differences do not fully account for sex-related variability in treatment response. Evidence demonstrating lower effective dose thresholds for dopamine D2 receptor occupancy in females treated with olanzapine, as well as sex-specific neurophysiological responses independent of plasma concentrations [30], supports the hypothesis of intrinsic central sensitivity differences. Hormonal modulation of dopaminergic and serotonergic pathways may partly explain these effects, reinforcing the concept that equivalent plasma concentrations do not necessarily translate into equivalent pharmacodynamic outcomes across sexes. Safety data further underscore the clinical relevance of these biological differences [33,34]. Pharmacovigilance analyses consistently report higher frequencies of dose-related ADRs in women receiving SSRIs, while women remain disproportionately affected by drug-induced long QT syndrome [31,33]. Endocrine-related adverse effects, including hyperprolactinemia, also appear more frequently in female patients treated with antipsychotics. At the same time, not all outcomes demonstrate uniform sex effects, and certain metabolic or weight-related changes show heterogeneous findings across studies [35]. This heterogeneity highlights the necessity of drug-specific evaluation rather than generalized sex-based assumptions. The interaction between age and sex emerges as a particularly important modifier. Older women consistently exhibit higher concentration-to-dose ratios for several antipsychotics, suggesting increased vulnerability to concentration-dependent toxicity in this subgroup. Despite this evidence, prescribing patterns frequently remain sex-neutral, even in vulnerable populations such as older adults in nursing home settings [36]. The absence of sex-informed dosing recommendations may therefore represent a missed opportunity to reduce preventable adverse events [37,49]. Taken together, the available evidence supports the inclusion of sex as a clinically meaningful variable in psychopharmacology, but it does not justify simplistic sex-based dose reductions across all agents. Instead, these findings argue for a stratified approach integrating sex, age, pharmacogenetic profile, and therapeutic drug monitoring into individualized treatment algorithms [38,53]. In this context, sex should be conceptualized as one component of a multidimensional precision framework rather than an isolated determinant [39,40]. This review has limitations. The included studies were heterogeneous in design, outcome definitions, and reporting formats, precluding quantitative meta-analysis. Pharmacovigilance data are inherently subject to reporting bias, and many clinical trials remain underpowered for robust sex-stratified analyses. Moreover, gender-related sociocultural factors—distinct from biological sex—were rarely examined, limiting conclusions regarding psychosocial modifiers of drug response [41,50]. While previous studies have independently explored sex differences in pharmacokinetics, pharmacodynamics, or adverse drug reactions, they have often done so in a fragmented manner, focusing on single drug classes or isolated outcomes [11,12,26]. In this context, the present review aimed to provide an integrated, clinically oriented synthesis of evidence across these domains, with the objective of identifying whether sex-related variability translates into actionable differences in psychotropic drug response. Compared to prior literature, our analysis highlights several critical issues in the current clinical framework. First, despite consistent signals of sex-related variability, these differences are rarely translated into sex-specific dosing recommendations or therapeutic strategies [12,53]. Second, many studies focus on selected parameters—such as pharmacokinetics or adverse event reporting—without integrating these findings into a comprehensive model of drug response [26]. Our synthesis reveals that this lack of integration may obscure clinically relevant patterns, particularly in terms of dose–exposure relationships and tolerability. At the same time, the heterogeneity observed across drug classes and outcomes suggests that sex differences are not uniform but rather drug- and endpoint-specific [35]. This represents both a limitation and an opportunity: while it challenges the development of universal sex-based guidelines, it also supports the potential for more precise, individualized therapeutic approaches [53]. Nevertheless, the convergence of pharmacokinetic, pharmacodynamic, and safety data across multiple independent sources strengthens the biological plausibility of sex-dependent variability in psychotropic drug response, reinforcing the need for a more systematic incorporation of sex as a clinical variable [16]. From a clinical perspective, these findings suggest that integrating sex-stratified data into prescribing practices could improve treatment tolerability and effectiveness, particularly in drugs with narrow therapeutic windows or significant metabolic burden. Future clinical trials should prospectively incorporate sex-stratified pharmacometric modeling and predefined dose–response analyses, to move from observational evidence to clinically actionable recommendations [38].

Another limitation of the present review is that the search was restricted to the PubMed database. While PubMed provides extensive coverage of biomedical literature, searching additional databases (e.g., Scopus or International Pharmaceutical Abstracts) could have increased the likelihood of identifying further relevant studies.

About the time period, we chose the most recent study about gender-based psychopharmacological treatments, so it is important to remember that the limited number of studies specifically addressing gender differences in psychopharmacotherapy should also be interpreted within a broader historical context. For several decades, women of childbearing age were often excluded from clinical trials due to concerns about potential risks during pregnancy and regulatory requirements regarding contraception in drug trials. Consequently, much of the early evidence supporting pharmacological treatments came primarily from male populations. These historical factors have contributed to persistent gaps in knowledge regarding sex-specific responses to psychotropic medications.

Additionally, regulatory frameworks may consider encouraging sex-specific exposure–response evaluations during drug development and post-marketing surveillance. Incorporating sex as a core variable in psychopharmacology aligns with the broader goals of personalized medicine: optimizing therapeutic efficacy while minimizing avoidable toxicity [42,43,44,45,46]. Translating these insights into clinical practice will require systematic integration of sex-informed evidence into dosing guidelines, monitoring strategies, and real-world prescribing algorithms [48,49,50,51].

## 5. Conclusions

Biological sex influences psychotropic drug exposure, receptor-level sensitivity, and adverse drug reaction profiles in clinically meaningful but drug-specific ways. Evidence from pharmacokinetic, pharmacodynamic, and pharmacovigilance studies indicates that females, particularly at older ages, may experience higher systemic exposure and differential pharmacodynamic responses to several SSRIs and second-generation antipsychotics. However, sex effects interact with age, genetic variability, and compound-specific characteristics, precluding uniform dose adjustments across all agents. Rather than supporting simplistic sex-based dosing rules, current evidence advocates for an integrated precision-medicine approach in which sex is considered alongside pharmacogenetics, therapeutic drug monitoring, and clinical risk profiling. Prospective trials incorporating predefined sex-stratified pharmacometrics analyses are needed to determine whether sex-informed dosing strategies can improve safety and therapeutic outcomes. Incorporating sex as a fundamental variable in psychopharmacology represents an essential step toward more individualized and safer psychiatric treatment.

## Figures and Tables

**Table 1:** Characteristics of primary studies investigating sex differences in psychotropic drug exposure, pharmacodynamics, and safety (*n* = 18): this table summarizes original investigations included in the qualitative synthesis, comprising pharmacokinetic studies, pharmacodynamic modeling analyses, observational cohorts, therapeutic drug monitoring datasets, pharmacovigilance database analyses, genetic association studies, and prescribing pattern investigations. For each study, drug class, study design, population, domain (PK, PD, or safety), and the main sex-specific findings relevant to personalized psychopharmacology are reported.

N.	Authors (Year)	Drug(S)/Class	Study Type	Study Design	Population/Setting	Domain (Pk/Pd/Safety)	Key Sex-Specific Finding Relevant to This Review
**1**	Cabaleiro et al. (2015) [28]	Risperidone	Primary	Pharmacokinetic + pharmacogenetic study	Adult patients	PK	CYP2D6 polymorphisms exert a stronger influence than sex; sex contributes to exposure variability
**2**	Calarge et al. (2014) [29]	Risperidone	Primary	Observational cohort	Children/adolescents	Safety (cardiometabolic)	Sex-stratified cardiometabolic outcomes reported
**3**	Ebina et al. (2025) [31]	Second-generation antipsychotics	Primary	Pharmacovigilance (JADER database)	National ADR reports	Safety	Sex-dependent reporting signals for dystonia
**4**	Ekhart et al. (2018) [32]	SSRIs	Primary	Pharmacovigilance analysis	National ADR database	Safety	Higher proportion of dose-related ADRs in women
**5**	Eugene & Masiak (2017) [35]	Olanzapine	Primary	Pharmacodynamic modeling study	Adults	PD	Females reach comparable D2 receptor occupancy at lower doses
**6**	Giménez et al. (2011) [36]	Olanzapine	Primary	Experimental single-dose study	Healthy volunteers	PD	Sex-specific sleep architecture modulation independent of plasma levels
**7**	Huang et al. (2026) [37]	Paroxetine	Primary	Model-informed precision dosing study	Patients with mental disorders	PK/Precision dosing	Sex included as a covariate in the individualized dosing model
**8**	Maclagan et al. (2020) [38]	Antipsychotics and benzodiazepines	Primary	Observational cohort	Nursing home residents with dementia	Safety/Prescribing	Sex differences in prescribing and exposure patterns
**9**	Morag et al. (2013) [39]	Antipsychotics	Primary	In vitro cellular sensitivity study	Human lymphoblastoid cells	PD (translational)	Sex-related differences in cellular drug sensitivity
**10**	Ramin et al. (2025) [40]	Antipsychotics	Primary	FDA adverse event database analysis	FAERS reports	Safety	Age- and sex-stratified adverse event profiles
**11**	Ramin et al. (2025) [41]	Antipsychotics	Primary	Claims database study	Outpatients with schizophrenia	Prescribing/Safety	Sex differences in outpatient prescribing patterns
**12**	Schulze et al. (2013) [42]	Antipsychotics	Primary	Prescription comparison study	Dementia vs. matched controls	Prescribing	Differential prescribing rates by sex
**13**	Seifert et al. (2021) [43]	Antidepressants	Primary	Pharmacovigilance program (AMSP)	2001–2017 database	Safety	Sex-related variability in antidepressant ADRs
**14**	Shi et al. (2012) [44]	Antidepressants	Primary	Genetic association study	Adult patients	PD/Treatment response	Female-specific genetic association with antidepressant response
**15**	Solhaug et al. (2025) [45]	Multiple antipsychotics	Primary	Therapeutic drug monitoring study (*n* ≈ 19,926)	Adult psychiatric patients	PK	Higher dose-adjusted serum concentrations in females; amplified with age
**16**	Sørup et al. (2020) [46]	Drugs for psychosis	Primary	Text-mining pharmacovigilance	Spontaneous reporting database	Safety	Sex-dependent adverse event reporting patterns
**17**	van der Horst et al. (2023) [47]	Clozapine	Primary	Observational study	Clozapine-treated patients	Safety + blood levels	Sex differences in ADR prevalence and blood-level dependency
**18**	Zang et al. (2021) [48]	Olanzapine	Primary	Population pharmacokinetic model	Chinese psychiatric patients	PK	Males exhibit higher apparent clearance than females

**Table 2:** Secondary evidence addressing sex differences in psychopharmacology (*n* = 10): This table summarizes systematic reviews, meta-analyses, narrative reviews, and clinical overviews that contextualize sex-related variability in psychotropic drug response and adverse reactions. These studies provide integrative or mechanistic perspectives supporting the interpretation of primary pharmacokinetic, pharmacodynamic, and safety data.

N.	Authors (Year)	Drug(S)/Class	Study Type	Study Design	Population/Setting	Domain	Key Sex-Specific Finding Relevant to This Review
**19**	Brand et al. (2022) [27]	Antipsychotics	Secondary	Narrative review	Women with schizophrenia	Clinical/Safety	Highlights the need for sex-informed antipsychotic prescribing
**20**	de Boer, S. (2023) [30]	Antipsychotics	Secondary	Narrative review	Female patients	Clinical/Safety	Provides practical sex-specific recommendations
**21**	Ercis et al. (2024) [33]	Mood stabilizers and antipsychotics	Secondary	Systematic review	Clinical populations	Efficacy/Safety	Reports sex-related variability in effectiveness and ADRs
**22**	Gurwitz (2013) [49]	Antipsychotics	Secondary	Review	Geriatric population	Safety	Discusses biological basis of sex bias in ADRs
**23**	Schoretsanitis et al. (2018) [50]	Olanzapine and risperidone	Secondary	Meta-analysis	Short- and mid-term trials	Safety (metabolic)	No consistent sex difference in weight gain
**24**	Sramek et al. (2016) [51]	Antidepressants	Secondary	Review	Adult patients	Clinical/Safety	Reviews sex differences in depression pharmacotherapy
**25**	Weersink et al. (2015) [52]	Antipsychotics	Secondary	Patient-reported outcome study	Adult consumers	Safety perceptions	Sex-related differences in safety concerns
**26**	Shan et al. (2023) [34]	Psychotropic, and cardiovascular, analgesic drugs	Secondary	Systematic review (26 studies)	Mixed populations	Safety (ADR-focused)	>50% evaluated ADRs show sex bias (e.g., lithium–thyroid in women; amisulpride–prolactin in women; clozapine–neutropenia in women)
**27**	Li et al. (2013) [53]	Multiple drugs (QT focus)	Secondary	Clinical review	Adults	Cardiac safety	Women at increased risk of drug-induced long QT syndrome

## Data Availability

The original contributions presented in this study are included in the article/Supplementary Materials. Further inquiries can be directed to the corresponding author.

## Supplementary Materials

The following supporting information can be downloaded at: https://www.mdpi.com/article/10.3390/jpm16040189/s1. PRISMA 2020 checklist.
